# *In Vitro* Anthelmintic Activity of Four Plant-Derived Compounds against Sheep Gastrointestinal Nematodes

**DOI:** 10.3390/vetsci5030078

**Published:** 2018-09-10

**Authors:** Federica Giovanelli, Matteo Mattellini, Gianluca Fichi, Guido Flamini, Stefania Perrucci

**Affiliations:** 1 Dipartimento di Scienze Veterinarie, University of Pisa, Viale delle Piagge 2, 56124 Pisa, Italy; federica.giovanelli76@gmail.com (F.G.); mattevet@yahoo.it (M.M.); gianluca.fichi@gmail.com (G.F.); 2 Dipartimento di Farmacia, University of Pisa, Via Bonanno Pisano 6, 56126 Pisa, Italy; guido.flamini@farm.unipi.it

**Keywords:** mangiferin, rutin, quercetin, β-sitosterol, anthelmintic activity, *in vitro*, gastrointestinal strongyles, sheep

## Abstract

By using the egg hatch test (EHT), the larval development test (LDT) and the larval mortality/paralysis test (LMT), the *in vitro* anthelmintic activity on sheep gastrointestinal strongyles (GIS) of four plant-derived pure compounds, mangiferin (at 0.25%, 0.125% and 0.0625%), rutin (at 1%, 0.75%, 0.5%), quercetin (at 1%), and β-sitosterol (at 1%, 0.75%, 0.5%), was investigated. For comparison, untreated and treated (0.1% thiabendazole, 0.1% TBZ) controls were used. Six repetitions were made throughout the experiment. Data were statistically elaborated using the χ^2^ test. The concentration able to inhibit the development of the 50% of L1s to L3s and causing the mortality of the 50% of L3s (EC50) was also calculated. L3s recovered from untreated Petri dishes were identified at the genus level. In EHT, all tested compounds at all concentrations significantly (*p* < 0.01) inhibited the hatch of the eggs when compared to the untreated controls, but none of them was as effective as 0.1% TBZ. In LDT, rutin (at 1%, 0.75% and 0.5%), mangiferin (at 0.25% and 0.125%), β-sitosterol (at 1%) and 0.1% TBZ completely prevented the larval development from L1 to L3 in respect to the untreated controls (*p* < 0.01). In LMT, all tested compounds significantly (*p* < 0.01) increased the death of L3s compared to the untreated controls, except for β-sitosterol at 0.5%. However, only rutin at all concentrations and 0.25% and 0.125% mangiferin were as effective as 0.1% TBZ. *Haemonchus*, *Trichostrongylus*, *Chabertia* and *Teladorsagia/Ostertagia* GIS genera, were identified.

## 1. Introduction

Gastrointestinal strongyles (GIS) are included among the most important factors affecting productivity and health of small ruminants worldwide [1,2,3,4]. GIS may limit sheep production by causing retarded growth, weight loss, reduced food consumption, lower milk production, impaired fertility and even mortality with heavy parasite burdens [2,5,6]. Strongyles infecting the digestive tract of small ruminants are a group of nematode species belonging to the order Strongylida. Their life cycle follows a similar pattern, in that adult nematodes live in the digestive tract of the host, where fertilised females produce eggs that are passed in the environment with the faeces of the host. After hatching, larvae (L1s) undergo two moults and develop to third-stage larvae (L3s) in the environment. Infection of small ruminants occurs mostly by ingestion of L3s that develop to mature adults in the digestive tract [7]. Although in recent years alternative approaches based on the use of several methods, such as grazing management, selective treatments, bioactive feed, biocontrol agents, animal selection for resistance to GIS and vaccination, have been developed [4,8,9,10,11,12,13], sheep GIS control programs still rely mostly on the use of synthetic anthelminthics [3,14,15,16]. However, the anthelmintic resistance developed by these nematodes worldwide has greatly limited the success of GIS control in sheep [1,3,6,17,18]. Indeed, some authors contend that anthelmintic resistance in sheep is the new standard and no longer the exception [6]. In the last 20 years, there has been considerable interest in the search for effective and safe herbal dewormers [5,19,20], and the use of effective plants and plant-derived compounds is now included among sustainable and targeted strategies for the control of these parasites [5,13,19,20]. After all, medicinal plants and plant extracts are used from a long time in ethnoveterinary medicine to treat farm animal diseases caused by helminths [13]. Moreover, studies on small ruminant behavior have revealed that these animals may self-medicated from helminth infections by selecting and ingesting specific plants [1]. In small ruminants, effective anthelmintic herbal compounds may increase profits by controlling GIS infections and GIS environmental contamination in pastures, and by reducing the use of conventional anthelmintics thus extending the useful life of the limited number of available anthelmintic drugs. Furthermore, the use of plant-derived compounds with anthelmintic properties is considered an effective method from the standpoint of GIS control for their potential lower environmental impact from residues in animal derived food products in relation to commercial anthelmintics [1,19,20,21].

In the present study, the *in vitro* anthelmintic activity of four plant-derived active principles, specifically mangiferin, rutin, quercetin and β-sitosterol, was investigated on sheep GIS. 

The choice of these herbal compounds was based on their anthelmintic activity *in vitro* and/or *in vivo* on nematodes reported in previous studies. More specifically, Garcia et al. (2003) [22] reported the *in vivo* anthelmintic activity of mangiferin on *Trichinella spiralis*. Rutin and quercetin demonstrated *in vitro* anthelmintic activity on sheep trichostrongyles in the studies of Barrau et al. [23] and of Kozan et al. [24], respectively. Finally, Villaseñor et al. reported the *in vitro* efficacy of β-sitosterol against *Ascaris suum* [25].

## 2. Materials and Methods 

### 2.1. Plant Materials

In the present study the *in vitro* anthelmintic activity of five plant-derived pure principles, including mangiferin (at 0.25%, 0.125% and 0.0625%), rutin (at 1%, 0.75% and 0.5%), quercetin (at 1%) and β-sitosterol (at 1%, 0.75% and 0.5%), was investigated on sheep GIS. 

All tested compounds were commercial products purchased from Sigma-Aldrich S.r.l. (Milan, Italy) and were diluted at different concentrations directly in the culture medium used for the *in vitro* cultivation of GIS eggs and larvae. The concentrations were expressed in percentage (weight/volume).

In order to select the most effective substances, also considering that the effectiveness of a compound is generally higher *in vitro* than *in vivo*, it was decided to continue the screening of each tested compound at lower concentrations, only if at the highest concentration tested some inhibiting effects were observed in all of the three assays performed in this study. 

### 2.2. Egg Recovery, Suspension and Cultivation of GIS Eggs and Larvae

Individual fresh faecal samples were collected from naturally infected ewes living in an organic sheep flock located in a natural reserve in which wild ruminants (mainly roe deer) lived. Ewes were positive for about 2000 GIS eggs per gram of faeces (EPG), after analysis with a McMaster method [26]. Recovery, suspension and cultivation of GIS eggs were performed within 3 h of collection by using previous reported methods [27,28]. For egg collection, faecal samples were pooled and about 10 g of faeces were mixed with 50 mL distilled water until a homogeneous suspensions was obtained. Each suspension was sieved and poured into a 50 mL tube and centrifuged at 900× *g* for 2 min. The supernatant was removed and the sediment was suspended in a saturated NaCl solution (specific weight 1.2), poured in 15 mL tubes and then centrifuged at 170× *g* for 2 min. Each supernatant containing GIS eggs was collected and eggs were washed with distilled water in 15 mL tubes, after centrifugation at 110× *g* for 2 min. The supernatant was removed and the sediment was collected. Then, 100 μL of the sediment were collected with a microtiter pipette, mounted on a glass slide and the number of eggs was microscopically counted. Batch of about 100 eggs were placed in sterile 6 cm Petri dishes and 3 mL of culture medium was added in each Petri dish. A total of 3 ml of culture medium contained: 0.54 mL of saline solution, 0.06 mL of Earl balanced salt solution (Sigma-Aldrich S.r.l., Milan, Italy), 12 μL of lyophilized *Escherichia coli* (*E. coli* strain W, lyophilized cells, Sigma-Aldrich S.r.l., Milan, Italy) suspended in water and sterilised for an hour at 100 °C, 12 μg of amphotericin B (Amphotericin B from *Streptomyces* approximately 80% HPLC, Sigma-Aldrich S.r.l., Milan, Italy), 60 mg of yeast extract (Sigma-Aldrich S.r.l., Milan, Italy) and about 2.4 mL of distilled water. The same culture medium and environmental conditions were used for larvae, by placing about 100 larvae/dish. Dishes were incubated in the dark at 24 °C for different periods according to the different tests, i.e., egg hatch test (EHT), larval development test (LDT) and larval mortality/paralysis test (LMT).

### 2.3. In Vitro Evaluation of the Anthelmintic Activity of Tested Compounds

For the *in vitro* evaluation of the anthelmintic activity of tested compounds, the egg hatch test (EHT), the larval development test (LDT) and the larval mortality/paralysis test (LMT), were used [29,30,31]. In all assays, tested compounds were diluted (weight/volume) at different concentrations directly in the culture medium and for each concentration six repetition were made. Petri dishes containing only the culture medium and Petri dishes containing the culture medium plus 0.1% thiabendazole (0.1% TBZ) (2-(4-Thiazoly) Benzimidazole minimum 99%, Sigma S.r.l. (Milan, Italy) were used as untreated and treated controls, respectively. TBZ was chosen due its relatively high solubility in water compared to other benzimidazoles [31]. Petri dishes were placed at 24 °C in a dark place. In EHT, culture Petri dishes containing about 100 GIS nematode eggs/Petri dish were prepared and microscopically checked after 48 h. First stage larvae (L1s) and unhatched eggs were then counted under the microscope and the percentages of hatched eggs were calculated. The LDT relies on the development of L1 to infective L3 larvae. In this test, about 100 GIS L1s/Petri dish were used. Petri dishes were checked after 7 days to evaluate the presence of third stage larvae (L3s). For each Petri dish, the number of L3s were counted under the microscope after adding a drop of Lugol’s iodine solution, and the percentages of L3s on the total number of larvae, were calculated. In LMT, about 100 live (motile) L3s/Petri dish were used. After 24 h, the percentage of L3s found motionless for at least 6 s at microscopical examination was calculated for each Petri dish. All data obtained from control untreated and treated cultures and from cultures treated with the different concentrations of the tested pure principles were statistically elaborated with the χ^2^ using Mc Graw Hill software [32]. In addition, for mangiferin, rutin, and β-sitosterol, the concentration (EC50; in %) able to inhibit the development in L3s of the 50% of L1s and causing mortality in the 50% of L3s was also calculated using Kärber’s formula [33]. 

### 2.4. Identification of GIS Nematode Genera

L3 recovered from LDT untreated controls were identified at the genus level based on their morphological and metric features [34] and the mean percentage of larvae of each identified genus on the total number of examined larvae was calculated. 

## 3. Results

In EHT (Table 1), 0.1% TBZ almost completely prevented the embryonation and hatching of GIS eggs. On the contrary, none of tested compounds was as effective as 0.1% TBZ (*p* < 0.01), since compared to TBZ, a significantly higher percentage of L1s was found in Petri dishes containing all tested pure compounds at all concentrations (Table 1). However, in this same assay all tested compounds gave results significantly different from those observed in the untreated controls (*p* < 0.01), except for 0.0625% mangiferin and 0.5% β-sitosterol. In LDT (Table 1), 0.25% and 0.125% mangiferin, rutin at all concentrations and 1% β-sitosterol completely prevented the larval development from L1 to L3, showing no statistical differences respect to 0.1% TBZ. Except for 1% quercetin that was ineffective, in LDT significant differences (*p* < 0.05) were found at statistical analysis in all other cases, both when compounds were compared to untreated and 0.1% TBZ treated controls. Except for 0.5% β-sitosterol (*p* < 0.05), in LMT (Table 1) larval mortality was really significantly higher (*p* < 0.01) in Petri dishes containing all tested active principles at all concentrations respect to that observed in the untreated controls (Table 1). However, among tested compounds only rutin at all concentrations and mangiferin at 0.25% and 0.125% were as effective as 0.1% TBZ, while statistical differences from TBZ treated controls were found in all other cases. Specifically, these differences were only significant (*p* < 0.05) in the case of 1% β-sitosterol and 0.0625% mangiferin, while they were highly significant (*p* < 0.01) in all other cases. The concentrations of 0.8%, 0.11% and 0.45% resulted the concentrations able to inhibit the 50% of larvae (EC50) in LDT and LMT assays found for β-sitosterol, mangiferin and rutin, respectively. *Haemonchus* (73%), *Trichostrongylus* (20%), *Chabertia* (5%) and *Teladorsagia*/*Ostertagia* (2%) were the GIS genera identified in sheep faecal samples used in this study.

## 4. Discussion

The severity of the disease caused by small ruminant GIS nematodes is mainly influenced by the parasite species present, environmental factors, management, nutrition, intensity of infection, age and general health and immunological status of the host [3]. Among this group of gastrointestinal nematodes, larvae identified in this study were found to belong mostly (73%) to the genus *Haemonchus* and to lesser extent to other genera, including *Trichostrongylus*, *Chabertia* and *Teladorsagia*/*Ostertagia* in descending percentage, respectively. *Haemonchus contortus* is the species of the genus *Haemonchus* infecting sheep and other livestock and it is considered one of the main parasites of small ruminants worldwide [35]. 

Among methods for the control of small ruminant GIS that rely less heavily on chemotherapy, the use of effective medicinal plants and plant-derived compounds is now considered a valid alternative [13,29]. Nonetheless, the anthelmintic efficacy of plant compounds is generally lower than that shown by synthetic drugs [13,29]. In small ruminants, plant-derived compounds can be used either as natural phytotherapeutic agents or as nutraceuticals, namely fodders or dietary supplements whose utilisation is associated with beneficial anthelmintic effects due to the presence in their composition of one or more active secondary metabolites [13]. For the evaluation of *the in vitro* anthelmintic activity on sheep GIS of the selected pure compounds, in the present study some tests considered valid for the evaluation of the *in vitro* efficacy of drugs on these helminths were used [30,31]. As expected, in the present study no tested plant compound showed an efficacy comparable to that of TBZ in all of the three assays performed. In particular, especially in the EHT, none of the pure compounds tested at all the concentrations used, showed the same effectiveness as TBZ. However, it is well known that anthelmintic drugs are not all equally able to prevent embryonation and hatching of nematode eggs, as this is a prerogative especially of benzimidazoles, while other anthelmintic drugs, as for example macrocyclic lactones, have no such ability [29,30,31,36]. This is the main reason why in studies evaluating the anthelmintic resistance of ruminant GIS strains, the EHT is used mainly for the detection of resistance to benzimidazoles [29,30,31,36]. Therefore, a reduced or absent efficacy of a molecule in this test should not necessarily be interpreted as indicative of low or absent anthelmintic properties. This is particularly true when they show to be highly effective in other kind of tests, as LDT, used to evaluate the anthelmintic resistance of these nematodes on a wider range of anthelmintic drugs, including macrocyclic lactones [36,37]. This is the case of most of the pure compounds examined in the present study. 

Among pure compounds considered in this study, rutin and quercetin are two principles from the flavonoid group of polyphenols that are found in many fruits, vegetables, leaves, and grains, being quercetin the aglycone component of rutin. In particular, rutin has been shown to contribute to the antibacterial properties of some invasive plant species [38]. In the present study, 1% rutin was the most effective compound in EHT, while at all concentrations tested (1%, 0.75% and 0.5%) it showed an efficacy comparable to that of 0.1% TBZ both in LDT and in LMT. On the contrary, 1% quercetin was ineffective in LDT and showed poor anthelmintic properties in EHT and LMT compared to rutin. Negative results of 1% quercetin in LDT is the main reason why it was not tested at lower concentrations. Results from the present study confirm the anthelmintic properties against *H. contortus* previously reported for rutin [23]. On the contrary, in the case of quercetin results obtained in the present study differ from those previously reported by Kozan et al. (2013) [24]. However, in this latter study quercetin was used at a higher concentration than that evaluated in the present study.

β-sitosterol is a sterol widely distributed in the plant kingdom showing a chemical structure similar to that of cholesterol. Although lower than that of rutin, in the present study this pure principle showed some efficacy in all assays (EHT, LDT and LMT) at the higher concentrations used (1% and 0.75%), while 0.5% β-sitosterol was ineffective. However, in EHT and LDT β-sitosterol was the pure principle that showed the most discordant results among replications, since high values of standard deviation were observed. In previous studies, β-sitosterol demonstrated *in vitro* antiprotozoal activity against *Giardia lamblia* and *Entamoeba histolitica* [39] and *in vitro* anthelmintic activity on *A. suum* [25].

Mangiferin is a plant xanthone for which a number of health-related properties have been reported [40], including antiparasite activity. In fact, Garcia and collaborators [22] found that oral administration of mangiferin (at 50 mg/kg once a day for 50 days) to rats experimentally infected with *Trichinella spiralis*, reduced both the number of encysted *T. spiralis* L1 larvae and specific anti-*Trichinella* IgE levels in the serum. Moreover, in a previous study this pure principle has been reported to be effective against *Cryptosporidium parvum* in experimentally infected mice [41]. In the present study, mangiferin was tested at very low concentrations (0.25%, 0.125% and 0. 065%) respect to the other compounds, because at higher concentrations the culture medium turned into gel. Nevertheless, in LDT and LMT this active principle at 0.25% and 0.125% showed an efficacy comparable to that of 1–0.5% rutin and of 1% and 0.75% β-sitosterol. Moreover, in LDT and LMT the anthelmintic activity of mangiferin at these two concentrations was comparable to that of the reference drug (TBZ). These interesting *in vitro* anthelmintic properties showed in LDT and LMT by concentrations of mangiferin much lower than that of the other compounds and comparable with that of the reference drug (TBZ), may be indicative that mangiferin was the most effective pure compound tested in the present study and confirm the anthelmintic properties previously reported for mangiferin [22]. 

The different *in vitro* anthelmintic activity of the various plant-derived compounds tested in this study may be explained, at least in part, by the different characteristics of their chemical structure. First, the different polarity of their molecules may be an important factor. In fact, from the comparison of the two related polyphenolic flavonoids rutin and quercetin, it can be observed that rutin is the most polar compound [42] and in this study it was also one of the most effective plant-derived substances. On the contrary, among polyphenols, i.e., mangiferin, rutin and quercetin, being comparatively the less polar of these polyphenols [43] quercetin showed the lowest efficacy. Thus, these results may be indicative that the different polarity may play an important role in the ability of these molecules to interact with GIS. However, in this study the polyphenol mangiferin was more effective than rutin, although its polarity is lower than that of rutin [40]. This could be explained by the different structure of its molecule respect to that of the other polyphenols tested in the present study, since mangiferin is a C-glucoside xanthone [40]. Therefore, the good in vitro anthelmintic efficacy of mangiferin observed in the present study could depend also on other factors related to its chemical structure that are worthy of further investigations. Although less polar of all other tested compounds, β-sitosterol at the higher concentrations showed an efficacy almost similar to that of rutin in LDT. Again, this could be explained by the different structure of its molecule because of a completely different biosynthesis in the plant. Indeed, polyphenols such as flavonoids or xanthones derive from a combination of the shikimate and acetate pathways [44,45]. On the contrary, sterols are modified triterpenoids, deriving from the mevalonate pathway [46,47]. Thus, the resulting molecules are very different from the previous ones and may possibly interact with different nematode structures.

## 5. Conclusions

Results obtained show, for the first time, that mangiferin and β-sitosterol are endowed with *in vitro* anthelmintic properties against sheep GIS. Previous reports on the effectiveness of rutin against *H. contortus* were confirmed in the present study and the anthelmintic properties of this pure principle against other gastrointestinal strongyle genera were demonstrated.

Plant pure principles tested in this study were found not equally effective on sheep gastrointestinal strongyles, as mangiferin and rutin showed higher anthelminthic properties. Therefore, mainly for these two compounds further studies aimed at evaluating their efficacy and toxicity *in vivo* are encouraged, especially with regard to their potential use as dewormers, as nutraceuticals or as a means to control the environmental development of gastrointestinal strongyles. Especially in the case of mangiferin, the *in vivo* activity against other parasite species reported for this compound in previous studies are promising about its possible efficacy on gastrointestinal strongyles also *in vivo*.

## Figures and Tables

**Table 1:** Sheep gastrointestinal strongyle egg hatch (EHT), larval development (LDT) and larval mortality/paralysis (LMT) observed in dishes containing mangiferin (at 0.25%, 0.125% and 0.0625%), rutin (at 1%, 0.75%, 0.5%), quercetin (at 1%), and β-sitosterol (at 1%, 0.75%, 0.5%) compared to untreated and treated (0.1% thiabendazole, TBZ) controls. Data are presented as percentages.

Treatment	EHT	sd	LDT	sd	LMT	sd
Untreated Controls	92.89	8.01	98.29	4.02	1.1	0.02
Tbz (0.1%) Controls	7.13	11.68	0	0	100	0
Mangiferin (0.25%)	58.28	15.6	0	0	100	0
Mangiferin (0.125%)	80.24	6.53	0	0	100	0
Mangiferin (0.0625%)	92.13	2.54	79.3	7.2	30.65	4.46
Quercetin (1%)	51.41	4.25	100	0	48.32	3.01
Rutin (1%)	23.71	6.35	0	0	100	0
Rutin (0.75%)	55.18	9.79	0	0	100	0
Rutin (0.5%)	71.53	9.04	0	0	100	0
β-sitosterol (1%)	42.74	29.48	1	2.2	80.28	5.1
β-sitosterol (0.75%)	44.74	30.36	8.02	11.74	50.09	10.35
β-sitosterol (0.5%)	100	0	93.01	8.53	6	12.97
